# Machine Learning Approach for Predicting the Impact of Food Insecurity on Nutrient Consumption and Malnutrition in Children Aged 6 Months to 5 Years

**DOI:** 10.3390/children11070810

**Published:** 2024-07-02

**Authors:** Radwan Qasrawi, Sabri Sgahir, Maysaa Nemer, Mousa Halaikah, Manal Badrasawi, Malak Amro, Stephanny Vicuna Polo, Diala Abu Al-Halawa, Doa’a Mujahed, Lara Nasreddine, Ibrahim Elmadfa, Siham Atari, Ayoub Al-Jawaldeh

**Affiliations:** 1 Department of Computer Sciences, Al Quds University, Jerusalem P.O. Box 20002, Palestine; 2 Department of Computer Engineering, Istinye University, 34010 Istanbul, Turkey; 3 Department of Nutrition and Food Technology, College of Agriculture, Hebron University, Hebron P.O. Box 40, Palestine; 4 Institute of Community and Public Health, Birzeit University, Ramallah P.O. Box 14, Palestine; 5 Nutrition Department, Ministry of Health, Ramallah P.O. Box 4284, Palestine; 6 Nutrition and Food Technology Department, Faculty of Agriculture and Veterinary Medicine, An-Najah National University, Nablus P.O. Box 7, Palestine; 7 Faculty of Medicine, Al-Quds University, Jerusalem P.O. Box 20002, Palestine; 8 Nutrition and Food Sciences Department, Faculty of Agriculture and Food Sciences, American University of Beirut, Beirut 1107 2020, Lebanon; 9 Department of Nutrition, Faculty of Life Sciences, University of Vienna, 1090 Vienna, Austria; 10 Regional Office for the Eastern Mediterranean, World Health Organization, Cairo 7608, Egypt

**Keywords:** food insecurity, malnutrition, wasting, stunting, machine learning, public health

## Abstract

Background: Food insecurity significantly impacts children’s health, affecting their development across cognitive, physical, and socio-emotional dimensions. This study explores the impact of food insecurity among children aged 6 months to 5 years, focusing on nutrient intake and its relationship with various forms of malnutrition. Methods: Utilizing machine learning algorithms, this study analyzed data from 819 children in the West Bank to investigate sociodemographic and health factors associated with food insecurity and its effects on nutritional status. The average age of the children was 33 months, with 52% boys and 48% girls. Results: The analysis revealed that 18.1% of children faced food insecurity, with household education, family income, locality, district, and age emerging as significant determinants. Children from food-insecure environments exhibited lower average weight, height, and mid-upper arm circumference compared to their food-secure counterparts, indicating a direct correlation between food insecurity and reduced nutritional and growth metrics. Moreover, the machine learning models observed vitamin B1 as a key indicator of all forms of malnutrition, alongside vitamin K1, vitamin A, and zinc. Specific nutrients like choline in the “underweight” category and carbohydrates in the “wasting” category were identified as unique nutritional priorities. Conclusion: This study provides insights into the differential risks for growth issues among children, offering valuable information for targeted interventions and policymaking.

## 1. Introduction

Food security deeply influences children’s health, impacting not only their current nutritional status but also their long-term cognitive, physical, and socio-emotional development [1,2]. Food insecurity (FI) is widely recognized as an indicator of a vast array of negative outcomes, such as undernutrition, overweight status, obesity, stress, depression, and poor academic performance, among the general population [3]. Indeed, the burden of FI on young children can be more dramatic than in adults, as food-insecure children are 140% more likely to develop iron-deficiency anemia and cognitive, mental, and psychomotor impairments [4]. Similarly, children in food-insecure households are more likely to present upper respiratory infections and to be hospitalized compared to their food-secure counterparts. 

Studies have shown varying effects of food insecurity on the weight status of children, indicating a need for more comprehensive analyses to understand the potential differential risks for growth issues [5,6,7,8]. Research presents different findings, with some studies noting a correlation between food insecurity and increased body weight in children, while others report no such link [3,5,9]. Additionally, certain investigations associate food insecurity with higher rates of obesity or overweight conditions in young children, whereas some studies suggest food insecurity may lead to malnutrition [6,7,8].

As for the causes of food insecurity, studies have shown that socioeconomic status plays a crucial role in influencing the outcomes of food insecurity and undernutrition [10,11]. Moreover, children living in food-insecure households often have an inadequate intake of macronutrients such as proteins, carbohydrates, and fats [12]. Protein–energy malnutrition, characterized by insufficient intake of protein and calories, is a common indicator of food insecurity in this age group [13]. This can lead to stunting, wasting, and an underweight status [14]. Micronutrient deficiencies are also prevalent among children in food-insecure environments. Essential vitamins and minerals, such as iron, vitamin A, iodine, and zinc, are often lacking in their diets. These deficiencies can lead to anemia, impaired cognitive development, weakened immune systems, and increased susceptibility to infections and diseases [15]. 

Indeed, food insecurity has been linked to undernutrition and stunting, among other forms of malnutrition, in children. The Joint Child Malnutrition Estimates (JME) report shows that an alarming 148.1 million children under the age of 5 experienced stunting in 2022, representing over 1/5 of children in this critical age group globally [16]. Moreover, Tiwari et al. highlighted a significant relationship between food insecurity and both stunting and severe stunting in children aged 0 to 59 months and 0 to 23 months, respectively, emphasizing the link between food insecurity and stunting in young children [11]. The debate continues about the extent to which FI influences the likelihood of undernutrition complications among children and adolescents.

Recently, machine learning (ML) algorithms, which combine elements of statistical learning and artificial intelligence research, are increasingly being utilized to analyze vast datasets, discovering hidden patterns or relationships and revealing the significance of predictors for specific problems [15,17,18]. Additionally, ML aids in the development of predictive models and the identification of the most crucial predictors [17,19]. There is a growing body of research utilizing ML for predicting various health conditions such as nutrition status, undernutrition, malnutrition, mortality, stunting, and anemia [15,17,20,21] using demographic and health survey datasets and nutrition risk factors.

Specifically, in the context of malnutrition, several studies have employed ML techniques. Talukder et al. used the Bangladesh Demographic and Health Survey (BDHS) 2014 data to predict malnutrition in children under five, finding that the Random Forest (RF) algorithm was the most effective [22]. Bitew et al. found that the Extreme Gradient (xgbTree) algorithm performed better using the Ethiopian Demographic and Health Survey 2016 data [23]. Khare et al. utilized the Indian Demographic Health Survey dataset 2005–2006 to explore correlations with malnutrition using artificial intelligence [24]. Furthermore, Shahriar et al. found that the Artificial Neural Network (ANN) was the most effective in classifying malnutrition among Bangladeshi children [25]. 

However, research on the application of machine learning (ML) algorithms to predict the relationship between food insecurity and malnutrition in children under five is limited. Therefore, this study aims to explore the impact of food insecurity and potential risk factors on child malnutrition and to classify its effects on children’s nutrient intake. Moreover, to the best of our knowledge, no study has yet investigated the link between food insecurity and different types of malnutrition (underweight, stunting, wasting, and undernutrition) to identify the nutrient factors most affected by each type of malnutrition. This research employs an ML approach to identify the nutrient factors most impacted by food insecurity, which will aid in developing more accurate models for predicting malnutrition risk, leading to more effective interventions and policies. Additionally, ML algorithms can offer data-driven insights by indicating the key predictors of malnutrition and providing valuable information to policymakers and public health officials for resource and intervention prioritization.

## 2. Materials and Methods

### 2.1. Data Source

This research was based on primary data collected from a cross-sectional study on food insecurity conducted in the West Bank, Palestine, in 2022. Our study assessed household food insecurity, nutritional status, nutrition awareness, attitudes, and parental practices. The sample comprised 1400 households, from which 819 children aged 6 months to 5 years (51.3% boys and 48.7% girls) were selected for this paper. 

In our ML analysis, we initially dealt with a small sample size of 819, leading to data imbalances. To address this, we applied the Synthetic Minority Over-sampling Technique (SMOTE), expanding our sample to 1226 participants [26]. This expansion aligns with the O = 2k heuristic for sample size calculation, ensuring a robust pool of subjects for analysis [27,28]. While some studies suggest needing up to 70k samples per variable for enhanced statistical power, our adjusted sample size strikes a balance between statistical rigor and the feasibility of identifying distinct clusters. SMOTE generated synthetic data by interpolating between neighboring instances in the minority class. We divided the dataset into four categories related to food insecurity: stunting, wasting, underweight, and undernutrition. Then, we used a 10-fold cross-validation method to evaluate model performance and minimize overfitting. SMOTE is widely used in fields like fraud detection and medical diagnostics.

Ethical clearance was granted by the Hebron University ethical committee on 17 October 2022, under reference number 17/7. Furthermore, informed consent was obtained from all participants before conducting the interviews.

This research focused on children from the West Bank aged 6 months to 59 months, with an average age of 33 months, including 52% boys and 48% girls. Those with disabilities or chronic conditions were not included in this study. Data were gathered using a structured questionnaire administered in person, which covered various personal, environmental, and dietary factors known to impact nutrition,. Sixteen trained research assistants carried out in-person surveys during home visits throughout all West Bank governorates.

### 2.2. Study Variables

The variables of this study were organized into four primary categories, detailed as follows:

1. Socioeconomic and Demographic Data: This part of the questionnaire aimed to gather information on social and environmental factors potentially impacting nutrition, such as geographical location, economic status, and household dynamics, as shown in Table 1. Typically, parents (usually mothers) provided the socioeconomic and demographic information for their households.
2. Household Food Insecurity: The Radimer/Cornell hunger scale, a 10-item questionnaire, was used to evaluate food insecurity at three levels: the household, the adult caregiver, and the child [29]. This scale captures various dimensions of food insecurity within the household.
3. Nutrition Status (Dietary Intake): Children’s nutrient intake was assessed using a 24 h dietary recall method [30]. A total of three 24 h recalls were collected for each child, including two on non-consecutive weekdays and one on a weekend day. This approach ensures a comprehensive representation of the children’s dietary intake across different days. Parents (mothers) reported all the food and beverages their children consumed in the previous 24 h, covering all meals (breakfast, lunch, and dinner) and snacks, including portion sizes and preparation methods. This method provided a comprehensive overview of each child’s daily dietary intake.
4. Anthropometric Measures: The research team recorded the height, weight, and mid-upper arm circumference (MUAC) of the study participants, with MUAC measurements specifically taken for older children. A portable SECA 217 body meter, equipped with a horizontal headboard, was used to measure height, with each measurement taken twice to ensure accuracy within 0.1 cm. Participants’ weight was measured using a SECA 874 digital scale, accurate to 0.1 kg, after they were asked to remove their shoes, socks, and any heavy clothing. These measurements were then converted into three indices using the WHO Anthro Software (Version 3, 2009): height-for-age Z-score (HAZ), weight-for-age Z-score (WAZ), and weight-for-height/length Z-score (WHZ). Based on these Z-scores, children under five were categorized into moderate and severe underweight, stunting, and wasting, defined as Z-scores below −2 and −3, respectively [31,32].

The MUAC was measured at the midpoint of the upper arm using a NutriActiva MUAC tape and recorded to the nearest 0.1 cm. Each child’s MUAC was measured twice, with the average value recorded as the final measurement. This value was then used to calculate the MUAC-for-Age Z-score (MUACZ) using the WHO Anthro Software (Version 3, 2009) [33,34]. Children were classified into moderate and severe acute undernutrition categories based on MUACZ scores below −2 and −3, respectively.

### 2.3. Nutrient Intake

Data from the 24 h dietary recall were analyzed using the nutrient analysis tool of the EMFID software Version 1. EMFID, established by Al Quds University in 2021 in collaboration with the World Health Organization (WHO), is a collaborative food database for Eastern Mediterranean countries. The software’s food composition tables converted the reported food and beverage consumption into their respective nutritional contents. This analysis provided information on the intake of macronutrients and micronutrients, such as energy, protein, carbohydrates, fats, fibers, B vitamins, vitamin C, vitamin A, and minerals like calcium (Ca), magnesium (Mg), potassium (K), phosphorus (P), copper (Cu), iron (Fe), and zinc (Zn). To ensure accuracy, daily energy and nutrient intake was calculated as the average intake from the two 24 h recalls.

The nutritional intake data were then evaluated against the Recommended Dietary Allowances (RDAs). The RDAs, developed and periodically updated by the U.S. National Research Council, serve as benchmarks for optimal nutrition [35]. They specify the necessary nutrient levels for children based on their age, gender, and anthropometric measurements, allowing for an assessment of whether their diets meet, exceed, or fall below the recommended levels.

### 2.4. Machine Learning Techniques

Machine learning (ML) techniques encompass a diverse array of algorithms, each with unique strengths and applications, particularly in the use of personalized precision health. These algorithms help identify complex patterns within data, thereby showing the potential of ML to advance areas such as personal weight management. This research looks at several machine learning methods, such as Support Vector Machine (SVM), Random Forest (RF), Logistic Regression (LR), Gradient Boosting (GB), and decision trees (DTs), and how they can be used in this field.

1. Support Vector Machine (SVM): SVM is a powerful algorithm used for both classification and regression tasks [36]. It works by finding the best boundary (or hyperplane) that separates data points from different categories with the widest margin. This boundary helps in classifying new data points into their respective categories. SVM is especially good at handling complex datasets where the relationship between data points is not straightforward, thanks to its ability to transform data into higher dimensions where data are easier to separate.
2. Random Forest (RF): Random Forest is an ensemble learning technique, which means it combines the predictions from multiple machine learning algorithms to make more accurate predictions than any individual model [37]. Specifically, RF builds multiple decision trees and merges their results. It is great for both classification and regression tasks. This approach helps in dealing with overfitting, which is a common problem with decision trees. Random Forest works well with large datasets and can handle both numerical and categorical data, making it versatile and robust.
3. Logistic Regression (LR): Logistic Regression is primarily used for binary classification problems—tasks that have two possible outcomes [38]. It predicts the probability that a given input point belongs to a certain class. It can predict the status of the target variable based on the set of associated features. LR works well with linearly separable data and is easy to implement and understand, making it a popular choice for many binary classification problems.
4. Gradient Boosting (GB): Gradient Boosting is a type of machine learning algorithm that improves predictions over time by combining the insights of several models, typically decision trees [39]. It starts with a base model and incrementally builds new models that correct the errors made by previous ones. This process continues until the model can no longer improve or reaches a specified number of trees. Gradient Boosting is effective for a wide range of tasks and can handle complex datasets with mixed types of data.

### 2.5. Model Validation and Performance Measures

This study adopted a detailed validation strategy along with multiple metrics to evaluate the model’s performance. The 10-fold cross-validation method was used for data validation, whereby the dataset is split into 10 parts, 80% of which is used for model training, while the remaining 20% is used for testing and validation, thus ensuring that each data point is tested once and enhancing model reliability by enhancing exposure to varied data scenarios.

For performance evaluation, the confusion matrix was utilized to visualize true and false positives and negatives. In addition, accuracy was utilized to measure the proportion of correct predictions. Given accuracy’s limitations in skewed datasets, precision was also considered given its importance for reducing false positives, as well as sensitivity (recall), crucial for identifying true positives. To balance precision and recall, the F1-score and the Fβ score (β = 0.5) were deployed to slightly favor precision.

Furthermore, the Kappa statistic assessed agreement levels, and the Area Under the Receiver Operating Characteristic (ROC) Curve, or AUC, measured the model’s class differentiation ability, with higher values indicating better performance. These metrics collectively provide a comprehensive view of the model’s effectiveness in distinguishing between different weight statuses.

The Random Forest ranking method was implemented to understand the relative importance of features in the model. This technique calculates the importance of each feature based on how much it decreases the impurity in the model’s decision trees. Features that contribute more to reducing impurity are ranked higher, providing insights into which factors are most influential in predicting malnutrition outcomes. This information is crucial for focusing interventions on the most impactful determinants of child malnutrition.

### 2.6. Data Analysis

The data were presented in two forms: continuous variables were shown as means and standard deviations (mean ± SD), including age (in months), weight (kg), height (cm), mid-upper arm circumference (MUAC in cm), and various Z-scores (e.g., weight-for-age Z-score, height-for-age Z-score); categorical variables were presented as frequencies and percentages (n, %), including sex (male/female), district (south/middle/north), locality (city/village/camp), family income (low/moderate/high), and household education level (≤secondary/university). The Shapiro–Wilk test was used to assess the normality of continuous variables, guiding the choice of subsequent statistical tests; the specific results of these tests were not detailed in the tables but were used to determine the appropriate methods. Differences between food-secure and food-insecure children in Table 2, Table 3, Table 4 and Table 5 were tested using univariate analysis, Mann–Whitney U tests, and chi-square tests. The level of significance was set at *p* ≤ 0.05 for all analyses, with *p* ≤ 0.001 indicating highly significant differences for variables showing particularly strong associations with food security status.

## 3. Results

### 3.1. Descriptive Analysis

Table 2 shows the descriptive and univariate analyses conducted to examine the link between food security and a range of sociodemographic and health factors. The findings revealed that out of 819 children surveyed, 18.1% were identified as food-insecure. Among the variables analyzed, five were particularly significant in their association with food insecurity. Household education emerged as the most critical determinant affecting food security, with family income, locality, district, and age following, respectively.

Indeed, 71.6% of food-insecure households have lower levels of household education, namely below secondary education (F-value: 258.7; *p* < 0.001), illustrating the impact of educational attainment on food security status. Similarly, economic status, as reflected in family income levels, shows a strong correlation with food insecurity, as 67.6% of the food-insecure group falls into the low-income category (F-value: 245.3; *p*-value: 0.001). Age groups, particularly infants (6–12 months) and toddlers between 36 and 48 months, show considerable variation in food insecurity (F-value: 7.4; *p* < 0.001), indicating age as a crucial factor. Geographic disparities are also significant, as evidenced by the substantial differences in food insecurity rates among districts, with the southern district exhibiting a notably higher rate of 53.4% (F-value: 11.3; *p* < 0.001). Moreover, the type of locality shows refugee camps expectedly reporting the highest rates of food insecurity (60.8%), a finding that is statistically significant with an F-value of 43.9 and a *p*-value of 0.001.

The results in Table 3 show the anthropometric measurements of children by household food security status. The results evidence that children in food-secure environments exhibited higher average weights and heights compared to their food-insecure counterparts, with significant differences in weight (13.93 ± 4.43 kg vs. 12.37 ± 5.04 kg, F-value: 7.3, *p* < 0.001) and height (87.48 ± 14.66 cm vs. 81.63 ± 16.45 cm, F-value: 6.5, *p* < 0.001). The mid-upper arm circumference (MUAC), an indicator of nutritional status, was also higher in food-secure children (16.61 ± 3.16 cm) compared to those classified as food-insecure (15.43 ± 2.71 cm, F-value: 3.1, *p* < 0.001).

Furthermore, the analysis investigated the Z-scores that measure growth and nutritional status against standardized growth charts. Food-secure children showed higher Z-scores across the results [33]. The weight-for-age Z-score (WAZ), height-for-age Z-score (HAZ), body mass index (BMI)-for-age, and MUAC-for-age Z-score (MUACZ) all indicated better nutritional and growth status in the food-secure group, with statistically significant differences observed in each measurement (*p* < 0.001).

The results in Table 4 show the univariate analysis of nutritional and weight status among children. In the underweight category, a significant observation is the presence of severe underweight status exclusively among the food-secure group (3.1%), with food-insecure children not reporting any severe cases. Likewise, moderate underweight status is marginally more prevalent in the food-secure group (1.5%) compared to the food-insecure group (0.7%) (F-value: 8.4, *p* = 0.004).

On the other hand, the “wasting” analysis indicates that moderate and severe wasting are notably higher among food-insecure children, with 6.8% experiencing moderate wasting and 1.4% experiencing severe wasting, compared to 3.3% and 0.6% in the food-secure group, respectively. This difference is statistically significant (F = 6, *p* = 0.015), indicating a stronger correlation between wasting and food insecurity.

The analysis in Table 5 shows significant associations between food insecurity and nutrient intake. Expectedly, the vast majority of nutrients presented a higher number of intake percentages below the Recommended Daily Allowance (RDA) in the food-insecure group compared to the food-secure group. Macronutrients such as proteins, carbohydrates, and fats evidenced a significant intake variation between food-secure and food-insecure groups. Food-insecure children had a significantly lower mean intake of these nutrients, indicating an association between food insecurity and an inadequate intake of essential macronutrients. The mean energy intake, in particular, greatly differed between food-secure (1218 ± 566.8 g) and food-insecure children (813.4 ± 555.3 g).

The intake of all micronutrients, including vitamin A, vitamin B1, vitamin B2, vitamin B3, vitamin B5, vitamin B6, vitamin B12, vitamin C, calcium, magnesium, manganese, phosphorous, potassium, copper, and zinc, was also significantly lower in the food-insecure group. Thus, food insecurity is not only affected by macronutrient intake but is also directly correlated to deficiencies in essential micronutrients.

The F-values and corresponding *p*-values indicate that the differences in nutrient intake between the food-secure and food-insecure groups are statistically significant for most nutrients. For instance, the differences in protein (F = 14.2, *p* < 0.001), carbohydrate (F = 16.4, *p* < 0.001), and fat (F = 16.4, *p* < 0.001) intake are all highly significant. Interestingly, the intake of folate, vitamin A, vitamin B3, vitamin B5, and phosphorus did not show a significant difference between the food-secure and food-insecure groups.

### 3.2. Machine Learning Analysis

The analysis in Table 6 evaluates the performance of four machine learning models, namely Random Forest (RF), Support Vector Machine (SVM), Gradient Boosting (GB), and Logistic Regression (LR), in predicting food insecurity and malnutrition among children aged 6 months to 5 years.

The Random Forest model exhibited the highest performance for the prediction of stunting, undernutrition, and wasting. The RF predicted stunting with significantly high levels of accuracy (0.977), AUC (0.996), F1 score (0.976), precision (0.977), recall (0.977), and MCC (0.920), yet closely matched by the GB model in most metrics. SVM and LR had lower performances, with Logistic Regression showing the lowest MCC (0.818). Likewise, the RF model performed the best for the prediction of undernutrition and wasting, followed by GB and SVM in both categories. Once again, LR showed significantly lower performance across all metrics.

However, the GB model exhibited the highest AUC (0.998) for the prediction of underweight status, closely followed by RF, with an AUC of 0.994. The RF model had the highest accuracy, F1 score, precision, recall, and MCC, with values of 0.986, 0.986, 0.986, 0.986, and 0.927, respectively. SVM and LR had lower performance compared to RF and GB, with Logistic Regression showing the lowest MCC (0.849).

The results showed that the RF model consistently outperformed all malnutrition indicators, followed closely by GB. SVM and LR generally have lower performance metrics. These results indicate that ensemble methods like RF and GB are highly effective in predicting food insecurity and malnutrition among children aged 6 months to 5 years.

The results in Table 7 show the analysis of several forms of malnutrition caused by food insecurity—stunting, underweight status, wasting, and undernutrition—across all four ML models (FI importance of nutrient factors alongside socioeconomic determinants and a precision view of malnutrition’s etiology).

Comparing the four models—FI-Stunting, FI-Underweight, FI-Wasting, and FI-Undernutrition—shows both common and distinct nutrient factors that emphasize the complex relationship between food insecurity and various forms of malnutrition. Indeed, sociodemographic factors such as family income and household education play an important role among all models, with locality showing similar relevance among stunting and undernutrition. Similarly, vitamin B1 emerges as a significant nutrient across all models, with particularly high importance in wasting (X^2^ = 196.0). Vitamin K1 also appears across several models, indicating its importance in overall growth and development. Vitamin A and zinc are other nutrients that persist across the different models.

However, each model also shows unique nutrient priorities that correspond to the specific malnutrition condition being analyzed. For instance, FI-Wasting emphasizes the need for vitamin B1 (X^2^ = 196.0), carbohydrates (X^2^ = 153.3), and magnesium (X^2^ = 147.2). Conversely, choline (X^2^ = 49.2), vitamin B1 (X^2^ = 48.1), and vitamin C (X^2^ = 44.8) show particular importance in the FI-underweight model. Meanwhile, the FI-Stunting model focuses on vitamins B1 (X^2^ = 49), K1 (X^2^ = 57), B3 (X^2^ = 48), A (X^2^ = 28.3), and Zinc (X^2^ = 27.2), reflecting the multifaceted nutritional needs to support long-term growth and prevent stunting.

FI-Undernutrition shows a broad range of nutrients, including vitamins B1 (X^2^ = 78.5), K1 (X^2^ = 48.3), and C (X^2^ = 44.4), copper (X^2^ = 44.4), and carbohydrates (X^2^ = 41.4), indicating the varied dietary requirements to combat the comprehensive challenges of undernutrition. Moreover, the ML ranking model shows that, while some nutrient deficiencies, such as vitamin B1, vitamin K1, vitamin A, and zinc, are universally critical in the fight against malnutrition caused by food insecurity, others like choline, carbohydrates, and magnesium have more targeted importance based on the specific malnutrition condition.

## 4. Discussion

This study has shown a high prevalence of food insecurity among children (18.1%), which is consistent with the findings of several studies that have explored food insecurity at the global level, particularly in regions with similar socioeconomic backgrounds [40,41]. This consistency shows the prevalent nature of food insecurity across diverse geographical and economic landscapes. The significant association of food insecurity with household education, family income, locality, district, and age showed the multifactorial nature of this issue, where socioeconomic determinants play a crucial role in shaping food security outcomes [42].

The linkage between household education and food security status could be attributed to the direct impact that educational attainment has on employment opportunities, income levels, and health literacy, all of which are essential for ensuring food security [43]. This finding aligns with the theory that education acts as a social determinant of health, influencing a wide range of health outcomes through its effects on economic and social conditions [44].

Economic status, as reflected in family income levels, showed a strong correlation with food insecurity, supporting the hypothesis that financial constraints limit access to adequate and nutritious food [10,42,45]. This relationship is well documented in the literature, where low-income households are often unable to afford the costs associated with a balanced diet, leading to food insecurity [42,46]. The impact of locality (particularly refugee camps) and district (with the southern district encompassing lower-income populations) on food security further suggests that environmental and infrastructural factors, such as access to markets and food distribution channels, play a critical role in determining food availability and accessibility [47].

Furthermore, the age-related variations in food insecurity observed in our study could be interpreted through the nature of lifecycle nutrition, where different age groups have unique nutritional needs and vulnerabilities. Younger children, in particular, are at a critical stage of growth and development, making them more susceptible to the adverse effects of food insecurity. This vulnerability indicates the importance of targeted nutritional interventions during early childhood to prevent long-term health consequences [48].

The findings regarding the nutritional and weight status of children provide empirical evidence supporting the detrimental impact of food insecurity on child health outcomes. The anthropometric measures indicate that food-insecure children suffer from malnutrition, as evidenced by lower average weights, heights, and MUACs compared to their food-secure counterparts. These differences in growth metrics can have serious implications for the physical and cognitive development of affected children, potentially leading to delayed growth and development, reduced academic performance, and increased susceptibility to infections and diseases [49].

The differences in nutrient intake between food-secure and food-insecure groups indicate the critical role of diet quality in determining health outcomes. The significantly lower intake of essential macronutrients and micronutrients among food-insecure children points to a diet lacking in diversity and nutritional adequacy, which is a common characteristic of food-insecure households. This nutritional inadequacy not only affects growth and development but also compromises immune function, increasing the risk of morbidity and mortality among affected populations [50].

The comparative analysis of machine learning models indicates significant insights into predicting malnutrition among children under five. The RF model consistently outperformed others across various malnutrition indicators such as stunting, undernutrition, wasting, and underweight, with remarkable metrics including an AUC of 0.996 for stunting and an MCC of 0.933 for undernutrition prediction. This superior performance is largely attributed to the ensemble approach of RF and GB, which synthesizes outcomes from numerous decision trees, thereby enhancing prediction accuracy and managing overfitting more effectively [17,51]. Conversely, SVM and LR showed lower efficacy, as demonstrated by LR’s lower MCC values. This discrepancy indicates the limitations of linear models like LR in capturing the complex, multidimensional interactions influencing malnutrition, a challenge better addressed by the more sophisticated ensemble methods [15,22,52].

Furthermore, this study explored the impact of food insecurity on malnutrition through machine learning models. In the analysis of FI-Stunting, the emergence of vitamin K1, vitamin B1, and vitamin B3 as critical factors affected by FI emphasizes their essential roles in child growth. This finding is corroborated by research indicating the pivotal importance of these vitamins in child growth and development [49]. Similarly, the impact of vitamin A on immune support and zinc on growth reinforces the consensus on their critical roles in child health [53].

The significant impact of choline, vitamin B1, and vitamin C among underweight children indicates the necessity of these nutrients for essential bodily functions such as liver function, energy metabolism, and nutrient absorption [54]. These findings are in line with studies highlighting the importance of a diverse and nutrient-rich diet in preventing underweight conditions among children [15,55].

The FI-Wasting model positions vitamin B1 at the forefront, emphasizing its importance for energy production and neural function, a perspective strongly supported by the literature on acute malnutrition management [56]. The importance of carbohydrates and magnesium for energy and muscle health, alongside vitamin K1, copper, and zinc for blood health and immune function, further aligns with nutritional strategies advocated for wasting treatment [57].

In FI-Undernutrition, the model identified vitamin B1 and vitamin K1, followed by copper, vitamin C, and carbohydrates, as key factors, indicating the broad spectrum of nutritional needs essential for combating undernutrition [58]. This is consistent with the integrated nutrition interventions recommended for addressing comprehensive nutritional challenges [58].

Comparatively, across the four models, vitamin B1’s significance in all forms of malnutrition shows its common importance in addressing FI-induced malnutrition. Vitamin K1’s recurrent appearance emphasizes its role in overall growth, while the consistent presence of vitamin A and zinc across models corroborates their known importance in child health. However, the distinct emphasis on specific nutrients like choline in FI-Underweight and carbohydrates in FI-Wasting points to the unique nutritional priorities necessitated by different malnutrition conditions.

## 5. Strengths and Limitations

This study presents a comprehensive analysis of the impact of food insecurity on children’s nutritional status, examining a range of sociodemographic factors, anthropometric measurements, and nutrient intake. It employs machine learning models to add a novel dimension to the analysis, enabling the identification of key predictors of malnutrition and offering insights for targeted interventions. This research also provides a detailed assessment of specific micronutrient intake, offering a precise understanding of nutritional deficiencies among food-insecure children.

However, this study’s cross-sectional design limits the ability to establish causal relationships between food insecurity and malnutrition. The reliance on self-reported data for household income and food security status may introduce bias and affect the accuracy of the findings. Additionally, the focus on children under five in the West Bank, Palestine, may limit the generalizability of the findings to other regions or age groups.

Regarding the 24 h recall method used to assess children’s nutrition, it is important to acknowledge its limitations. This method depends on participants’ memory and accuracy, which can introduce recall bias and lead to underreporting or overreporting of food intake. It captures only a single day’s intake, which may not reflect usual dietary patterns, especially in children whose daily intake can vary. Additionally, the food composition database may not have complete nutrient profiles for all foods, potentially leading to an underestimation of certain nutrient intakes. Despite these limitations, the use of machine learning models in our study helps identify key predictors and provides valuable insights into malnutrition among food-insecure children.

## 6. Conclusions

This study shows the profound impact of food insecurity on the nutritional status of children aged 6 months to 5 years in the West Bank, Palestine. Food insecurity is linked to a lower intake of essential nutrients, resulting in poorer growth metrics and an increased risk of malnutrition. Machine learning models have identified key nutrient factors influenced by food insecurity, providing valuable insights for the development of targeted interventions. Addressing the complex determinants of food insecurity and ensuring adequate nutrient intake are essential for enhancing child health and preventing malnutrition. This research adds to the growing evidence supporting a comprehensive approach to combating malnutrition, indicating the importance of addressing both macro- and micronutrient deficiencies in food-insecure populations.

## Figures and Tables

**Table 1:** Study variables.

Section	Items
Socioeconomic and Demographic Data	Sex, age, administrative district, geographic region (southern, central, and northern), locality (refugee camp, village, or city), household education level, household size, employment status of the father, employment status of the mother, and household income.
Household Food Insecurity	Food quantity, food quality, food acceptability, and the certainty of obtaining food.
Nutrition Status (Dietary Intake)	Recall (24 h): grams intake, energy, protein, carbs, fiber, fats, vitamin B1 (thiamine), vitamin B2 (riboflavin), vitamin B3 (niacin), vitamin B5, vitamin B6, choline, vitamin B9 (folate), vitamin B12, vitamin C, vitamin E, vitamin K1, calcium, magnesium, phosphorous, potassium, sodium, copper, iron, manganese, and zinc.
Nutrition Status (Anthropometric Measures)	Length, weight, mid-upper arm circumference, and body mass index (BMI).

**Table 2:** Demographic and socioeconomic characteristics of children aged 6 months to 5 years by household food security status.

Variables		Food-Secure n = 671 (n%)	Food-Insecure n = 148 (n%)	Total 819 (n%)	F (*p*-Value)
Age (months)	6–12	106 (15.8)	41 (27.7)	147 (17.9)	7.4 **
	12–24	135 (20.1)	37 (25)	172 (21)	
	24–36	152 (22.7)	12 (8.1)	164 (20)	
	36–48	138 (20.6)	38 (25.7)	176 (21.5)	
	48–60	140 (20.9)	20 (13.5)	160 (19.5)	
Sex	Male	340 (50.7)	80 (54.1)	420 (51.3)	0.56
	Female	331 (49.3)	68 (45.9)	399 (48.7)	
**District**	South	298 (44.4)	79 (53.4)	377 (46)	11.3 **
	Middle	150 (22.4)	48 (32.4)	198 (24.2)	
	North	223 (33.2)	21 (14.2)	244 (29.8)	
**Locality**	City	255 (38)	8 (5.4)	263 (32.1)	43.9 **
	Village	230 (34.3)	50 (33.8)	280 (34.2)	
	Camp	186 (27.7)	90 (60.8)	276 (33.7)	
**Family Income**	Low	49 (7.3)	100 (67.6)	149 (18.2)	245.3 **
	Moderate	257 (38.3)	38 (25.7)	295 (36)	
	High	365 (54.4)	10 (6.8)	375 (45.8)	
**Household Education**	≤Secondary	106 (15.8)	106 (71.6)	212 (25.9)	258.7 **
	University	565 (84.2)	42 (28.4)	607(74.1)	

** statistically highly significant: *p*-value ≤ 0.001.

**Table 3:** Anthropometric measurements of children aged 6 months to 5 years by household food security status (n = 819).

Variables	Food-Secure n = 671 (n%)	Food-Insecure n = 148 (n%)	F-Value (*p*-Value)
Weight	13.93 ± 4.43	12.37 ± 5.04	7.3 (0.001) **
Height	87.48 ± 14.66	81.63 ± 16.45	6.5 (0.001) **
MUAC	16.61 ± 3.16	15.43 ± 2.71	3.1 (0.001) **
Weight-for-age	0.4 3± 1.26	0.08 ± 1.95	12.8 (0.001) **
Height-for-age	−0.7 ± 2.38	−1.07 ± 2.6	9.3 (0.001) **
Weight-for-height	1.4 ± 2.49	1.15 ± 1.91	9.1 (0.001) **
MUACZ	0.72 ± 2.11	−0.03 ± 1.94	8.8 (0.001) **

** *p* < 0.001; MUAC: mid-upper arm circumference; MUACZ: mid-upper-arm-circumference-for-age Z-score.

**Table 4:** Nutritional and weight status of children aged 6 months to 5 years by household food security status (n = 819).

		Food-Secure n = 671 (n%)	Food-Insecure n = 148 (n%)	Total n = 819 (n%)	F-Value (*p*-Value)
Underweight	Normal	640 (95.4)	147 (99.3)	787 (96.1)	8.4 (0.004) *
	Moderate	10 (1.5)	1 (0.7)	11 (1.3)	
	Severe	21 (3.1)	0 (0)	21 (2.6)	
Wasting	Normal	645 (96.1)	136 (91.9)	781 (95.4)	6 (0.015) *
	Moderate	22 (3.3)	10 (6.8)	32 (3.9)	
	Severe	4 (0.6)	2 (1.4)	6 (0.7)	
Stunting *	Normal	510 (76)	103 (69.6)	613 (74.8)	5.3 (0.023) *
	Moderate	86 (12.8)	25 (16.9)	111 (13.6)	
	Severe	75 (11.2)	20 (13.5)	95 (11.6)	
Undernutrition	Normal	615 (91.7)	134 (90.5)	749 (91.5)	17.8 (0.001) **
	Moderate	29 (4.3)	6 (4.1)	35 (4.3)	
	Severe	27 (4)	8 (5.4)	35 (4.3)	

* Statistically significant: * *p* < 0.05; ** *p* < 0.001.

**Table 5:** Association between food insecurity and nutrient intake among Palestinian children aged 6 months to 5 years in the West Bank.

Nutrient (Unit)	Nutrient Intake per RDA		Food Security Level		
	≥RDA n (%)	<RDA n (%)	Food-Secure Mean ± SD	Food-Insecure Mean ± SD	F (*p*-Value)
Energy (kcal)	432 (52.7)	387 (47.3)	1218 ± 566.8	813.4 ± 555.3	14.9 **
Protein (g)	295 (36)	524 (64)	44.6 ± 24.9	30 ± 23	14.2 **
Carb (g)	110 (13.4)	709 (86.6)	162.5 ± 77.1	110.2 ± 74.2	16.2 **
Fat (g)	787 (96.1)	32 (3.9)	45.3 ± 26.2	29.3 ± 22.9	16.4 **
Fiber (g)	735 (89.7)	84 (10.3)	11.1 ± 7.7	6.8 ± 7.3	10.1 **
Folate (mg)	434 (53)	385 (47)	175.7 ± 163.5	112.9 ± 130.4	0.7
Vit A (mg)	298 (36.4)	521 (63.6)	159.2 ± 219	78.2 ± 165.6	0.8
VitB1 (mg)	465 (56.8)	354 (43.2)	1.3 ± 2	0.4 ± 1	6 *
VitB2 (mg)	287 (35)	532 (65)	3.5 ± 4	2.2 ± 2.9	12.4 **
VitB3 (mg)	172 (21)	647 (79)	7.1 ± 5.4	5 ± 6.3	2.5
VitB5 (mg)	308 (37.6)	511 (62.4)	12.4 ± 41.5	11.9 ± 57.4	0.2
VitB6 (mg)	493 (60.2)	326 (39.8)	2 ± 3	0.6 ± 0.4	17.3 **
VitB12 (mcg)	316 (38.6)	503 (61.4)	2 ± 2.5	1.8 ± 2.7	4.9 *
Vit C (mg)	817 (99.8)	2 (0.2)	46.1 ± 46.1	29.7 ± 35.5	26.1 **
Ca^1^ (mg)	513 (62.6)	306 (37.4)	467 ± 317.3	307.6 ± 257.1	3.2 *
Mg^2^ (mg)	614 (75)	205 (25)	125.6 ± 74.6	100.8 ± 77	70.1 **
Mn^3^ (mg)	264 (32.2)	555 (67.8)	1.9 ± 3.2	1.5 ± 2.9	11.6 **
P^4^ (mg)	341 (41.6)	478 (58.4)	562.6 ± 321.5	436 ± 332.1	1.3
K^5^ (mg)	711 (86.8)	108 (13.2)	1190.8 ± 619.2	847.3 ± 546.5	4.5 *
Cu^6^ (mg)	180 (22)	639 (78)	1.4 ± 1.9	1.3 ± 2.5	6.8 *
Fe^7^ (mg)	441 (53.8)	378 (46.2)	8.2 ± 6.7	5 ± 5.3	8 **
Zn^8^ (mcg)	379 (46.3)	440 (53.7)	5.34.2	3.93.5	23.1 **

* Statistically significant: * *p* < 0.05; ** *p* < 0.001; Ca^1^: calcium, Mg^2^: magnesium, Mn^3^: manganese, P^4^: phosphorous, K^5^: potassium, Cu^6^: copper, Fe^7^: iron, and Zn^8^: zinc.

**Table 6:** ML models performance analysis in predicting food insecurity and malnutrition among children aged 6 months to 5 years.

Model		AUC	Accuracy	F1	Precision	Recall	MCC
Stunting	RF	0.996	0.977	0.976	0.977	0.977	0.920
	SVM	0.957	0.965	0.963	0.966	0.965	0.878
	GB	0.996	0.976	0.975	0.975	0.976	0.916
	LR	0.975	0.947	0.947	0.946	0.947	0.818
Undernutrition	RF	0.992	0.968	0.968	0.968	0.968	0.933
	SVM	0.968	0.940	0.940	0.940	0.940	0.875
	GB	0.978	0.942	0.942	0.942	0.942	0.880
	LR	0.891	0.804	0.804	0.804	0.804	0.593
Wasting	RF	0.996	0.972	0.972	0.973	0.972	0.945
	SVM	0.982	0.953	0.953	0.954	0.953	0.907
	GB	0.988	0.951	0.951	0.951	0.951	0.901
	LR	0.937	0.874	0.874	0.874	0.874	0.747
Underweight	RF	0.994	0.986	0.986	0.986	0.986	0.927
	SVM	0.977	0.981	0.980	0.981	0.981	0.900
	GB	0.998	0.985	0.985	0.985	0.985	0.921
	LR	0.990	0.971	0.971	0.971	0.971	0.849

MCC is Matthews Correlation Coefficient; AUC is Area Under the Curve.

**Table 7:** ML models’ feature importance ranking by malnutrition form (FI-Stunting, FI-Underweight, FI-Wasting, and FI-Underweight).

FI-Stunting		FI-Underweight (n = 819)		FI-Wasting (n = 819)		FI-Undernutrition (n = 819)	
Factor	X^2^	Factor	X^2^	Factor	X^2^	Factor	X^2^
Family Income	145.4	Family Income	134.1	Vitamin B1	196.0	Family Income	108.3
Education	75.9	Education	84.2	Education	164.6	Locality	79.1
Locality	75.5	Choline	49.2	Carbs	153.3	Vitamin B1	78.5
Vitamin K1	57.0	Vitamin B1	48.1	Magnesium	147.2	Education	49.1
Vitamin B1	49.0	Vitamin C	44.8	Vitamin K1	139.6	Vitamin K1	48.3
Vitamin B3	48.0	Locality	39.9	Copper	85.6	Copper	44.4
Vitamin A	28.3	Vitamin K1	28.4	Age	57.9	Vitamin C	44.4
Zinc	27.2	Vitamin A	23.5	Zinc	51.4	Carbs	41.4
Vitamin C	24.5	Sodium	17.5	Vitamin A	41.1	Zinc	39.1
Carbs	23.4	Vitamin B3	17.2	Fat	34.4	Age	30.1
Choline	20.9	Fat	15.1	Choline	30.8	Vitamin B3	29.9
Copper	20.4	Vitamin B6	15.0	Vitamin B12	27.9	Vitamin A	23.9
Age	18.1	Protein	14.9	Locality	27.6	Protein	23.5
Magnesium	16.0	Zinc	14.7	Gender	26.0	Choline	18.2
Fat	15.1	Copper	10.1	Iron	21.4	Folate	12.7
Protein	8.7	Gender	7.6	Calcium	16.8	Fat	11.8
Calcium	6.3	Vitamin B12	7.4	Manganese	16.6	Magnesium	11.6
Iron	5.3	Vitamin B2	5.8	Potassium	12.3	Vitamin B12	9.7
Vitamin B6	4.7	Magnesium	5.2	Protein	4.2	Vitamin B6	9.3
Phosphorus	3.2	Phosphorus	5.1	Vitamin B3	3.1	Vitamin B5	8.1

FI: food insecurity; X^2^: chi-squared.

## Data Availability

Data are available upon request from the authors. The data are not publicly available due to need of conducting more analysis on this data from the study authors.

## References

[1] Moradi S., Mirzababaei A., Mohammadi H., Moosavian S.P., Arab A., Jannat B., Mirzaei K. (2019). Food insecurity and the risk of undernutrition complications among children and adolescents: A systematic review and meta-analysis. Nutrition.

[2] Rahman S.M.J., Ahmed N.A.M.F., Abedin M., Ahammed B., Ali M., Rahman J. (2021). Maniruzzaman Investigate the risk factors of stunting, wasting, and underweight among under-five Bangladeshi children and its prediction based on machine learning approach. PLoS ONE.

[3] Garcia J., Hromi-Fiedler A., E Mazur R., Marquis G., Sellen D., Lartey A., Pérez-Escamilla R. (2013). Persistent household food insecurity, HIV, and maternal stress in Peri-Urban Ghana. BMC Public. Health.

[4] Pai S., Bahadur K. (2020). The Impact of Food Insecurity on Child Health. Pediatr. Clin. N. Am..

[5] Berra W.G. (2020). Household Food Insecurity Predicts Childhood Undernutrition: A Cross-Sectional Study in West Oromia (Ethiopia). J. Environ. Public. Health.

[6] Disha A., Purnima M., Rahul R. (2013). Household food insecurity is associated with higher child undernutrition in bangladesh, ethiopia, and vietnam, but the effect is not mediated by child dietary diversity. J. Nutr..

[7] Mulu E., Mengistie B. (2017). Household food insecurity and its association with nutritional status of under five children in Sekela District, Western Ethiopia: A comparative cross-sectional study. BMC Nutr..

[8] Rosen F., Settel L., Irvine F., Koselka E.P.D., Miller J.D., Young S.L. (2024). Associations between food insecurity and child and parental physical, nutritional, psychosocial and economic well-being globally during the first 1000 days: A scoping review. Matern Child Nutr..

[9] Abdalla L., Goulao L.F. (2024). Food security and nutrition in refugee camps in the European Union: Development of a framework of analysis linking causes and effects. Food Secur..

[10] Kang Y., Kim J. (2019). Risk factors for undernutrition among children 0–59 months of age in Myanmar. Matern. Child. Nutr..

[11] Tiwari R., Ausman L.M., Agho K.E. (2014). Determinants of stunting and severe stunting among under-fives: Evidence from the 2011 Nepal Demographic and Health Survey. BMC Pediatr..

[12] Troen A.M., Fraser D., Abdeen Z., Rosenberg I.H. (2006). Child Nutrition Initiative in Israel and Palestine: Status of food security, micronutrient malnutrition, and behavioral change and communication programs. Food Nutr. Bull..

[13] Denney J.T., Brewer M., Kimbro R.T. (2020). Food insecurity in households with young children: A test of contextual congruence. Soc. Sci. Med..

[14] Basiry M., Surkan P.J., Ghosn B., Esmaillzadeh A., Azadbakht L. (2024). Associations between nutritional deficiencies and food insecurity among adolescent girls: A cross-sectional study. Food Sci. Nutr..

[15] Gebeye L.G., Dessie E.Y., Yimam J.A. (2023). Predictors of micronutrient deficiency among children aged 6–23 months in Ethiopia: A machine learning approach. Front. Nutr..

[16] UNICEF, WHO World Bank Group Levels and Trends in Child Malnutrition. https://iris.who.int/bitstream/handle/10665/368038/9789240073791-eng.pdf?sequence=1.

[17] Gustriansyah R., Suhandi N., Puspasari S., Sanmorino A. (2024). Machine Learning Method to Predict the Toddlers’ Nutritional Status. J. INFOTEL.

[18] Cruz A.D., Gallegos N.I., Gattud K.A., Antonio V.A., Miro E.D., Go C.C. (2024). Using machine learning algorithms to determine the food insecurity level of households of public school children. AIP Conference Proceedings.

[19] Saroj R.K., Yadav P.K., Singh R., Chilyabanyama O. (2022). Machine Learning Algorithms for understanding the determinants of under-five Mortality. BioData Min..

[20] Gao J., Lu Y., Domingo I., Alaei K., Pishgar M. (2024). Predicting Sepsis Mortality Using Machine Learning Methods. medRxiv.

[21] Qasrawi R., Badrasawi M., Abu Al-Halawa D., Polo S.V., Abu Khader R., Al-Taweel H., Abu Alwafa R., Zahdeh R., Hahn A., Schuchardt J.P. (2024). Identification and prediction of association patterns between nutrient intake and anemia using machine learning techniques: Results from a cross-sectional study with university female students from Palestine. Eur. J. Nutr..

[22] Talukder A., Ahammed B. (2020). Machine learning algorithms for predicting malnutrition among under-five children in Bangladesh. Nutrition.

[23] Bitew F.H., Sparks C.S., Nyarko S.H. (2022). Machine learning algorithms for predicting undernutrition among under-five children in Ethiopia. Public. Health Nutr..

[24] Khare S., Kavyashree S., Gupta D., Jyotishi A. (2017). Investigation of Nutritional Status of Children based on Machine Learning Techniques using Indian Demographic and Health Survey Data. Procedia Computer Science.

[25] Shahriar F., Roy B., Datta S.D. Mechanical properties of polypropylene fiber reinforced high-strength concrete exposed to elevated temperatures: An experimental and artificial neural network approach. Proceedings of the 7th International Conference on Civil Engineering for Sustainable Development (ICCESD).

[26] Kovács B., Tinya F., Németh C., Ódor P. (2020). SMOTE: Synthetic Minority Over-sampling Technique Nitesh. Ecol. Appl..

[27] Jiang K., Lu J., Xia K. (2016). A novel algorithm for imbalance data classification based on genetic algorithm improved SMOTE. Arab. J. Sci. Eng..

[28] Xu Z., Shen D., Nie T., Kou Y., Yin N., Han X. (2021). A cluster-based oversampling algorithm combining SMOTE and k-means for imbalanced medical data. Inf. Sci..

[29] Kendall A., Olson C.M., Frongillo E.A. (1995). Validation of the Radimer/Cornell measures of hunger and food insecurity. J. Nutr..

[30] Huang K., Zhao L., Fang H., Yu D., Yang Y., Li Z., Mu D., Ju L., Li S., Cheng X. (2022). A Preliminary Study on a Form of the 24-h Recall That Balances Survey Cost and Accuracy, Based on the NCI Method. Nutrients.

[31] WHO Anthro Survey Analyser and Other Tools. https://www.who.int/tools/child-growth-standards/software.

[32] Myatt M., Khara T., Schoenbuchner S., Pietzsch S., Dolan C., Lelijveld N., Briend A. (2018). Children who are both wasted and stunted are also underweight and have a high risk of death: A descriptive epidemiology of multiple anthropometric deficits using data from 51 countries. Arch. Public Health.

[33] Sullivan K.M., Gorstein J. (1999). ANTHR: Software for Calculating Anthropometry Division of Nutrition and Physical Activity National Center for Chronic Disease Prevention and Health Promotion Centers for Disease Control and Prevention USA and the Department of Nutrition for Health and Development.

[34] Abitew D.B., Yalew A.W., Bezabih A.M., Bazzano A.N. (2021). Comparison of Mid-Upper-Arm Circumference and Weight-For-Height Z-Score in Identifying Severe Acute Malnutrition among Children Aged 6-59 Months in South Gondar Zone, Ethiopia. J. Nutr. Metab..

[35] National Research Council, Commission on Life Sciences, Food and Nutrition Board (1989). Subcommittee on the Tenth Edition of the Recommended Dietary Allowance. Recommended Dietary Allowances.

[36] Pisner D.A., Schnyer D.M. (2019). Support vector machine. Machine Learning: Methods and Applications to Brain Disorders.

[37] Pathak Y.P., Prakash I., Dholakia M.B., Professor A. (2023). Conventional and modern approaches in landslide susceptibility mapping: A methodological review. Int. J. Appl. Eng. Technol..

[38] Bisong E. (2019). Building Machine Learning and Deep Learning Models on Google Cloud Platform.

[39] Natekin A., Knoll A. (2013). Gradient boosting machines, a tutorial. Front. Neurorobot..

[40] Pereira A., Handa S., Holmqvist G. (2021). Estimating the prevalence of food insecurity of households with children under 15 years, across the globe. Glob. Food Secur..

[41] Pereira A.L., Handa S., Holmqvist G. (2017). Prevalence and Correlates of Food Insecurity among Children across the Globe. Ann. Ist. Super. Sanita..

[42] Mansur M., Afiaz A., Hossain S. (2021). Sociodemographic risk factors of under-five stunting in Bangladesh: Assessing the role of interactions using a machine learning method. PLoS ONE.

[43] Owoo N.S. (2021). Demographic considerations and food security in Nigeria. J. Soc. Econ. Dev..

[44] Carletto C., Zezza A., Banerjee R. (2013). Towards better measurement of household food security: Harmonizing indicators and the role of household surveys. Glob. Food Sec.

[45] Anku E.K., Duah H.O. (2024). Predicting and identifying factors associated with undernutrition among children under five years in Ghana using machine learning algorithms. PLoS ONE.

[46] Khan J.R., Tomal J.H., Raheem E. (2021). Model and variable selection using machine learning methods with applications to childhood stunting in Bangladesh. Inform. Health Soc. Care.

[47] Carter M.A., Dubois L., Tremblay M.S. (2014). Place and food insecurity: A critical review and synthesis of the literature. Public. Health Nutr..

[48] Bell L.K., Golley R.K. (2015). Interventions for Improving Young Children’s Dietary Intake through Early Childhood Settings: A Systematic Review. Int. J. Child Health Nutr..

[49] Black R.E., Allen L.H., Bhutta Z.A., Caulfield L.E., de Onis M., Ezzati M., Mathers C., Rivera J., Maternal and Child Undernutrition Study Group (2008). Maternal and child undernutrition: Global and regional exposures and health consequences. Lancet.

[50] Food Security and Nutrition (2020). The World the State of Transforming Food Systems for Affordable Healthy Diets.

[51] Saberi-Karimian M., Khorasanchi Z., Ghazizadeh H., Tayefi M., Saffar S., Ferns G.A., Ghayour-Mobarhan M. (2021). Prediction of malnutrition in newbornInfants using machine learning techniques Prediction of malnutrition in newborn Infants using machine learning techniques. Crit. Rev. Clin. Lab. Sci..

[52] Islam M., Rahman J., Islam M., Roy D.C., Ahmed N.F., Hussain S., Amanullah M., Abedin M. (2022). Maniruzzaman Application of machine learning based algorithm for prediction of malnutrition among women in Bangladesh. Int. J. Cogn. Comput. Eng..

[53] Caulfield L.E., De Onis M., Blössner M., Black R.E. (2004). Undernutrition as an underlying cause of child deaths associated with diarrhea, pneumonia, malaria, and measles 1–3. Am. J. Clin. Nutr..

[54] Morris A.L., Mohiuddin S.S. (2023). Biochemistry, Nutrients.

[55] Dewey K.G., Mayers D.R. (2011). Early child growth: How do nutrition and infection interact?. Matern. Child Nutr..

[56] Collins S., Dent N., Binns P., Bahwere P., Sadler K., Hallam A. (2006). Management of severe acute malnutrition in children. Lancet.

[57] Bhutta Z.A., Ahmed T., Black R.E., Cousens S., Dewey K., Giugliani E. (2008). What works? Interventions for maternal and child undernutrition and survival. Lancet.

[58] Ruel M.T., Alderman H. (2013). Nutrition-sensitive interventions and programmes: How can they help to accelerate progress in improving maternal and child nutrition?. Lancet.

